# Social behavior and decision making in bacterial conjugation

**DOI:** 10.3389/fcimb.2014.00054

**Published:** 2014-04-29

**Authors:** Günther Koraimann, Maria A. Wagner

**Affiliations:** ^1^Institute of Molecular Biosciences, University of GrazGraz, Austria; ^2^Max von Pettenkofer-Institut, Ludwig-Maximilians-Universität MünchenMunich, Germany

**Keywords:** horizontal gene transfer (HGT), gene regulatory networks, mobile genetic elements, bacterial conjugation, integrative conjugative elements (ICE), conjugative plasmids (CP)

## Abstract

Bacteria frequently acquire novel genes by horizontal gene transfer (HGT). HGT through the process of bacterial conjugation is highly efficient and depends on the presence of conjugative plasmids (CPs) or integrated conjugative elements (ICEs) that provide the necessary genes for DNA transmission. This review focuses on recent advancements in our understanding of ssDNA transfer systems and regulatory networks ensuring timely and spatially controlled DNA transfer (*tra*) gene expression. As will become obvious by comparing different systems, by default, *tra* genes are shut off in cells in which conjugative elements are present. Only when conditions are optimal, donor cells—through epigenetic alleviation of negatively acting roadblocks and direct stimulation of DNA transfer genes—become transfer competent. These transfer competent cells have developmentally transformed into specialized cells capable of secreting ssDNA via a T4S (type IV secretion) complex directly into recipient cells. Intriguingly, even under optimal conditions, only a fraction of the population undergoes this transition, a finding that indicates specialization and cooperative, social behavior. Thereby, at the population level, the metabolic burden and other negative consequences of *tra* gene expression are greatly reduced without compromising the ability to horizontally transfer genes to novel bacterial hosts. This undoubtedly intelligent strategy may explain why conjugative elements—CPs and ICEs—have been successfully kept in and evolved with bacteria to constitute a major driving force of bacterial evolution.

## Introduction

Bacterial conjugation is important not only for bacterial evolution, but also for human health since it represents the most sophisticated form of HGT in bacteria and provides, for instance, a platform for the spread and persistence of antibiotic resistance genes (Norman et al., 2009). To efficiently counteract the problems associated with antibiotic resistance it is therefore necessary to understand the mobile genetic elements—conjugative plasmids (CPs) and integrative conjugative elements (ICEs)—that are the vehicles for transfer of antibiotic resistance genes from the large communal gene pool to human pathogenic bacteria. In the following sections we will give an overview on the current knowledge of bacterial conjugation. As will be evident, it is a widely distributed, if not ubiquitous phenomenon in the bacterial world. Special emphasis will be given to regulatory mechanisms ensuring timely and spatially controlled expression of *tra* genes. Furthermore, we consider recent advancements in understanding population dynamics and coevolution of CPs and host cells. In the context of this manuscript intelligence is understood as cell-cell communication and complex regulatory systems producing cellular responses that maximize successful DNA transmission and at the same time do not impose a burden (or fitness cost) to the whole population of CP carrying cells.

### Bacterial conjugation modules

Bacterial conjugation is a cell-cell contact dependent DNA transfer event. Either dsDNA or ssDNA molecules are transported from donor to recipient bacterial cells. The transfer of dsDNA depending on one single dedicated protein (an FtsK like ATPase) is found only in Actinobacteria (Vogelmann et al., 2011; Thoma and Muth, 2012) and will not be considered further in this review. ssDNA transfer on the other hand is ubiquitous in the bacterial and archebacterial world and relies on a dedicated cell envelope spanning DNA transfer machinery ancestral to T4SS (type IV secretion systems) which translocate virulence determining effector proteins into target eukaryotic cells (Bhatty et al., 2013; Guglielmini et al., 2013). Approximately 10–20 proteins (fewer in Gram positive bacteria, see below) constitute the building blocks of the T4SS dedicated to ssDNA and protein transfer. The T4S machinery and additional proteins required for DNA transfer and replication (De la Cruz et al., 2010) are encoded by CPs or ICEs (Smillie et al., 2010; Guglielmini et al., 2011). Other genetic elements such as mobilizable plasmids or genomic islands can be mobilized by either of these self-transmissible elements (Smillie et al., 2010; Puymège et al., 2013). Unlike in true bacterial conjugation where DNA is transferred directly from a donor to a recipient cell, *Neisseria gonorrhoeae* secretes ssDNA contact-independently via a T4SS encoded by a genomic island (Ramsey et al., 2011).

### Conjugative plasmids (CPs) and integrated conjugative elements (ICEs)

**Figure 1:**
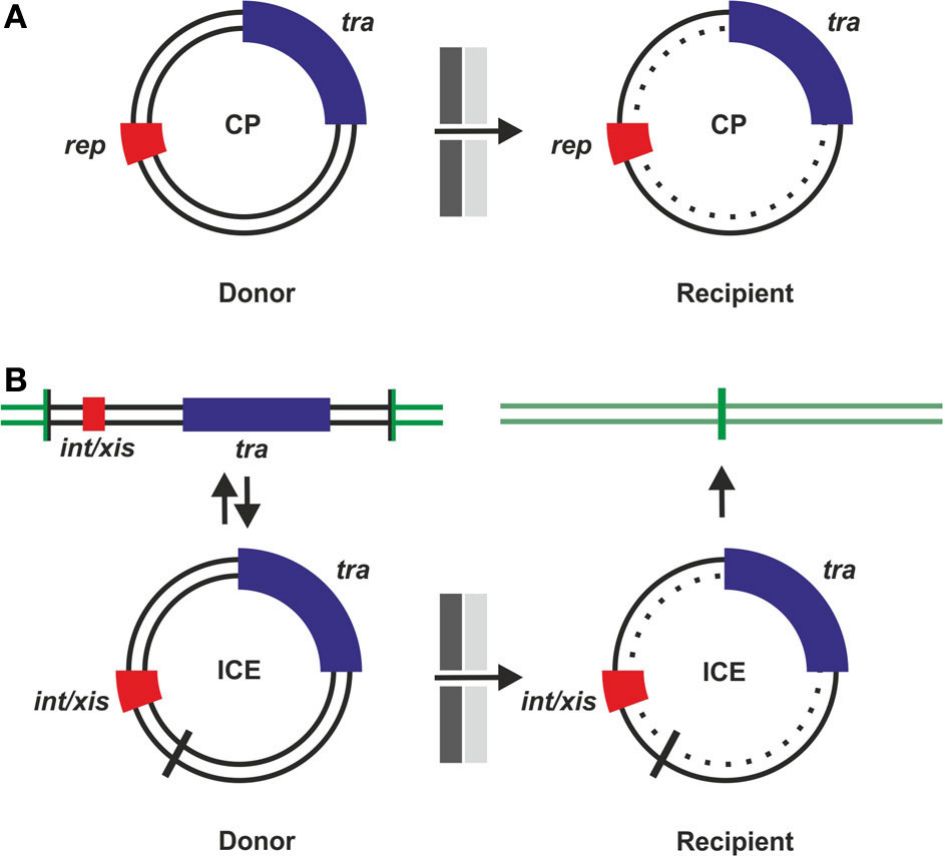
**Conjugative plasmids (CPs) and integrative conjugative elements (ICEs)**. Events leading to horizontal transfer of CPs **(A)** or ICEs **(B)** are shown schematically. Before transfer can occur, *tra* genes must be expressed and a T4SS assembled. After cell-cell contact formation, transfer competent donor cells initiate a rolling circle type replication from circular dsDNA and translocate ssDNA via the T4S machinery into recipient cells. dsDNA is then reconstituted in the recipient (dotted inner circle). **(A)** CPs can autonomously replicate due to the presence of *rep* genes. **(B)** ICEs replicate as integrated elements with the host chromosome (green lines), integration and excision is mediated by *int/xis* genes required for integration and excision by site-specific recombination via attachment sites (vertical bars). After excision and before integration, ICEs are present in a plasmid-like dsDNA form.

Historically, research on bacterial conjugation focused on the F-plasmid and related CPs from Gram negative bacteria (Willetts and Skurray, 1980; Frost et al., 1994). The interest shifted to broad host range CPs such as RP4 and R388 which encode a DNA transfer system more similar to the T-DNA transfer machinery encoded by *virB* genes of Ti (tumor inducing) plasmids of *Agrobacterium tumefaciens* (Eisenbrandt et al., 1999; Gomis-Rüth et al., 2001; Schröder and Lanka, 2003; Cascales and Christie, 2004). For structural studies on the T4S machinery plasmid pKM101 has had a pivotal role since it was possible to determine the 3D structure of a ring like core T4S complex composed of 14-mers of three proteins which span the periplasmic space from the inner to the outer membrane (Rivera-Calzada et al., 2013). From genomic sequencing projects and bioinformatics analyses it became evident that the most abundant self transmissible elements are ICEs that are maintained chromosomally similarly to temperate bacteriophages and can be transferred via a plasmid intermediate (Wozniak et al., 2009; Guglielmini et al., 2011). A schematic comparison of how CPs and ICEs are maintained and transferred is depicted in Figure 1.

### ssDNA transfer in gram positive and gram negative bacteria

ssDNA transfer through T4S machineries has been explored in detail for Gram positive and Gram negative bacteria and excellent reviews describing and comparing these systems have been published recently (Bhatty et al., 2013; Goessweiner-Mohr et al., 2013). Only a subset of proteins typically found in Gram negative bacteria is also present in the Gram positives which led to the concept of minimized T4SS that are present in Gram positive bacteria (Zhang et al., 2012; Bhatty et al., 2013; Goessweiner-Mohr et al., 2013). Major differences arising from the specific architecture of the cell envelope of diderms vs. monoderms are: The presence of a more complex T4SS spanning two membranes (including the periplasm and a thin peptidoglycan layer) with a cell-surface attached filamentous pilus composed of multiple subunits of a single protein in Gram negative bacteria; a minimized T4SS for translocating ssDNA across the cytoplasmic membrane with a dedicated peptidoglycan hydrolase for local digestion of the thick cell wall and adhesins that mediate cell-to-cell contact in Gram positive bacteria (Bhatty et al., 2013; Goessweiner-Mohr et al., 2013).

### Components and functions of ssDNA transfer machines

ssDNA is generated in the donor cell by proteins that can initiate a rolling circle type replication by nicking (cleaving) one strand of the dsDNA at a site termed *oriT* (origin of transfer). The nucleoprotein complex consists of the nicked plasmid DNA and the proteins required for DNA transfer and replication (also termed Dtr, usually a relaxase/ helicase and auxiliary proteins). Presumably, at this stage, the Dtr complex is docked to the T4S complex which has been pre-assembled in the cell envelope (Zechner et al., 2012). The T4S complex consists of (i) ATPases fueling assembly of the T4S apparatus and DNA transfer, (ii) translocon proteins of the inner membrane, (iii) core proteins spanning the cell envelope, and (iv) pilus proteins or adhesins. The Dtr complex physically interacts with the T4S apparatus mainly via protein-protein interactions especially via one of the ATPases of the T4S complex being a substrate receptor (Bhatty et al., 2013).

In order to start translocating ssDNA, a productive and stable mating pair between a donor and a suitable recipient cell has to be formed. This includes initial contact via the pilus or adhesins, pilus retraction in F conjugation (Clarke et al., 2008), and the formation of larger contact zones that have been observed in different conjugation systems (Dürrenberger et al., 1991; Samuels et al., 2000; Lawley et al., 2002). It is not known whether the pilus additionally functions as a device delivering ssDNA by penetration of the recipient cell envelope. Upon an elusive signal, ssDNA with the relaxase covalently bound to the 5 prime end of the ssDNA is transported through the conjugation channel (the T4S apparatus) and reaches the cytoplasm of the recipient where the DNA is recircularized (presumably via the co-transported relaxase) to regenerate a circular ssDNA which can be replicated to dsDNA in the recipient (Zechner et al., 2012). Establishment of the ds plasmid DNA in the recipient is aided by ssDNA binding, anti-restriction and SOS inhibition proteins, usually encoded by “leading region” genes which are among the first to enter the recipient cell (Althorpe et al., 1999; Wilkins, 2002). Overall, conjugative DNA replication is similar to the replication of ssDNA phages in which ssDNA (in that case termed the plus strand) is generated by rolling circle replication from a dsDNA intermediate, packaged into viral proteins and then released into the environment, ready to infect novel recipient cells.

Transfer of plasmid DNA into cells already containing the same CP is prevented by blocking cell-to-cell contact formation and entry of the ssDNA into the recipient cell, mechanisms that are termed “surface exclusion” and “entry exclusion,” respectively (reviewed in Garcillán-Barcia and de la Cruz, 2008).

## How and when to turn DNA transfer genes on

**Figure 2:**
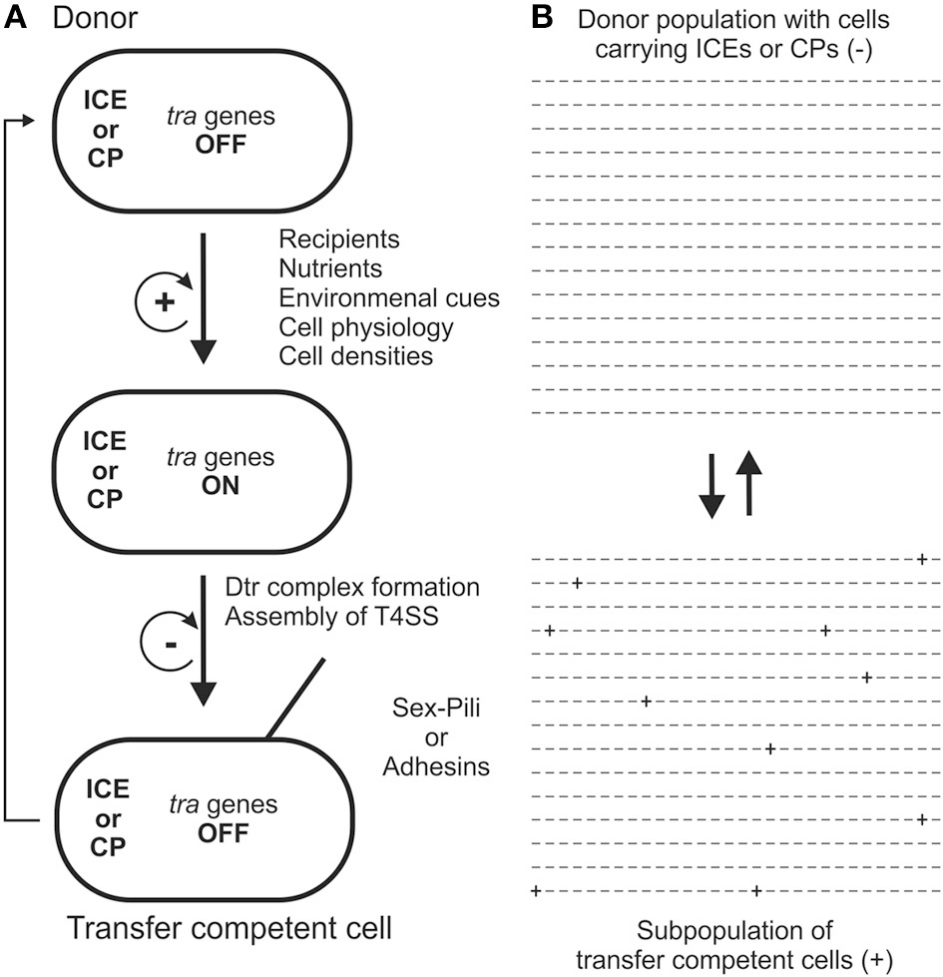
**Two models for the development of transfer competence in single cells (A) and populations (B) are shown**. In single cells **(A)** *tra* genes are turned ON by a variety of stimuli. A positive feedback loop ensures that, once initiated, cells proceed to transfer competence, involving formation of the Dtr complex and assembly of the T4S apparatus. In transfer competent cells, *tra* genes are switched OFF, mediated by a negative feedback loop. Eventually, transfer competence is lost by transition to unfavorable conditions. In unstructured, well-mixed populations **(B)** only a few donor cells (indicated by + signs) develop transfer competence, thereby minimizing the fitness cost for the population. For examples, detailed descriptions and discussions, the reader is referred to the section “How and when to turn DNA transfer genes ON” in the main text of this review.

Strategies to secure successful gene transfer in natural environments require sensing mechanisms that ensure that *tra* genes (Dtr and T4S genes) are turned ON at the right time and at the right place. There are several well studied systems which demonstrate that intricate regulatory networks evolved in different transfer systems to minimize metabolic burden and cellular stress, the threat of being attacked by “male” specific bacteriophages yet at the same time maximizing successful gene transfer via conjugation (Frost and Koraimann, 2010). As a general rule, the default status of transfer genes is OFF. Sensing specific signaling molecules (indicating the presence of recipients or high cell densities) and/or environmental conditions (e.g., nutrients, oxygen, temperature) is generally required to induce transfer gene expression in a subset of the donor cell population. Transfer gene expression followed by assembly of the T4S machinery in the cell envelope and formation of the Dtr complex transforms donors into transfer competent cells. Molecular regulatory networks and switches coupled to positive feedback loops ensure that once a certain threshold (which is defined at the single cell level) is reached, *tra* genes are expressed and the system is turned ON. Negative feedback loops ensure that—after the expression burst—the system returns to the default OFF state (for a general scheme see Figure 2A). At the molecular level, transcription of *tra* genes is controlled by repressor proteins and/or depends on activators. Some of the well-studied conjugation systems that display these features, with a focus on how *tra* genes are turned ON, are described in the following sections.

## Recipient sensing

### Recipient sensing through donor secreted inhibitory peptides

In ICE*bs*1, an ICE of the Gram positive bacterium *Bacillus subtilis*, transfer can be stimulated by the presence of recipient cells. Recipient sensing by donors is achieved through a processed and secreted inhibitory peptide, termed PhrI, that is then transported back into the cells. If only donors carrying ICE*bs*1 are present, at high cell densities, PhrI blocks an activator, RapI. When ICE*bs*1 free cells are around—which also take up PhrI—RapI can activate *int*/*xis* (for excision of the ICE), *tra* genes and subsequent conjugative transfer (Auchtung et al., 2005) in the ICE*bs*1 positive cells. RapI activates by stimulating a specific anti-repressor protease ImmA which negatively controls the ImmR repressor required to maintain the OFF state (Bose and Grossman, 2011). A similar case with a Rap protein counteracting an Xre-type transcriptional repressor exists in case of the native *B. subtilis* conjugative plasmid pLS20 (Singh et al., 2013). In both examples, the presence of recipients is sensed by an inhibitory peptide that is secreted by cells containing the conjugative element. A requirement for this system to work is that the recipient cells surrounding donors possess an uptake system for the secreted inhibitory peptide, thereby reducing its concentration in donors.

### Recipient sensing through recipient secreted conjugation pheromones

A variation of this theme is present in the two well-studied pheromone-inducible CPs of *Enterococcus faecalis*, pAD1 and pCF10, respectively. Recipient cells are sensed through a secreted, chromosomally encoded peptide pheromone (cAD1/cCF10). The peptide pheromone is actively transported into the cytosol of donor cells where it counteracts the inhibitory peptide (iAD1/iCF10) and inactivates the repressor (TraA/PrgX) followed by transcription of Dtr and T4S genes (reviewed in Clewell, 2011; Dunny and Johnson, 2011). As in many other cases, the repressor (TraA of pAD1) does not control *tra* genes directly, but an activator protein which, when it escapes repressor control, is positively auto-activated and efficiently transforms the donor cell into the transfer competent state (Clewell, 2011). If donor cell densities are too high, the inhibitory quorum sensing molecule iCF10 keeps donor cells in the OFF state. Similarly, iCF10 is also responsible for shutting off the *tra* genes after an initial burst caused by the inducing factor (Chatterjee et al., 2013).

## Sensing environmental conditions and host cell physiology

### Repressor inactivation mediated by the SOS response

Similarly to the temperate bacteriophage lambda, ICEs contain integrases and excisionases for integration and excision (*int*/*xis* in Figure 1B). In SXT, an ICE of *Vibrio cholerae*, a repressor protein with similarity to the lambda CI repressor, SetR, maintains the OFF status of the integrated conjugative element. The repressor can be inactivated by RecA mediated autocleavage through DNA damaging agents which induce the SOS response. Repressor inactivation is followed by expression of SetC and SetD which act as activators of *int*/*xis* and *tra* genes (Beaber et al., 2004). The low transfer frequency observed for SXT transfer and repressor inactivation is presumably maintained by a subpopulation of cells that inherently express SOS genes (McCool et al., 2004), specific inducers of this system, however, are unknown.

### Activation by specific nutrients and quorum sensing

Agrobacteria harboring Ti plasmids are not only capable of transforming plant cells by T-DNA transfer but also contain a specific set of DNA transfer genes for conjugation. Tra genes are not transcribed unless a specific transcriptional activator, TraR is expressed. First of all, *traR* transcription is dependent on opines, amino sugars specifically produced by transformed plant tumor cells. Opines can be taken up and used as nutrients only by agrobacteria harboring the Ti plasmid. Opines, specifically nopalines, inactivate a Ti plasmid encoded repressor (AccR) that controls several genes on the Ti plasmid. Among the genes controlled by AccR is the gene for the transcriptional activator TraR. Secondly, TraR acts as a receptor for—and is additionally activated by—an N-acyl-L-homoserine lactone (AHL) quorum signaling molecule. A positive feedback loop is constituted by the fact that production of AHL by TraI is also under the control of TraR. As a consequence, Ti plasmid encoded *tra* genes are only turned ON inside crown galls (where opines are produced by plant cells) at high cell densities (reviewed in White and Winans, 2007). In the Ti plasmid system, induction of *tra* genes is therefore dependent on signal molecule mediated repressor inactivation and activator production, which, once initiated, is enhanced by a positive feedback loop (provided by AHL synthesis), presumably resulting in a burst of *tra* gene expression in individual cells harboring the Ti plasmid. The system can be turned OFF by anti-activators (TraM and TrlR under the control of TraR—negative feedback loop) and may be modulated by lactonases that can specifically hydrolyze the AHL molecule in response to plant signals (Haudecoeur and Faure, 2010). Besides the Ti plasmid and two chromosomes, *Agrobacterium tumefaciens* C58 also harbors another large conjugative plasmid, pAT. Interestingly, conjugation genes of pAT are activated by opines but are independent of AHL (Lang et al., 2013).

### Activator escape and environmental cues in F-like plasmids

Notwithstanding the lack of obvious signaling molecules involved in F-conjugation module mediated DNA transfer, sensing environmental conditions in combination with the physiological status of the potential donor cell affects the behavior of the cell through a network of regulatory elements (for a detailed description see Frost and Koraimann, 2010). CPs with F-like conjugation modules are mainly found in the Enterobacteriaceae including pathogenic *Escherichia*, *Salmonella*, and *Klebsiella* species. Typically, the plasmid encoded transcriptional activator of *tra* genes, TraJ, is under the negative control of two fertility inhibition elements, FinO and FinP. While FinP is a small regulatory RNA that is produced as a countertranscript to the translation initiation region of the TraJ mRNA, FinO is an RNA chaperone that is required for efficient suppression of TraJ expression (Arthur et al., 2003). In populations of donor cells—under optimal conditions promoting growth and cell division—only in few cells (1–10 out of 1000 potential donor cells) TraJ escapes this negative FinOP mediated control and promotes transcription of *tra* genes together with the host encoded transcriptional activator ArcA-P (Strohmaier et al., 1998; Frost and Koraimann, 2010; Wagner et al., 2013). Once initiated, a positive feedback-loop leads to a burst of *tra* gene expression which ensures the transformation into a transfer competent cell (Dempsey, 1989; Pölzleitner et al., 1997). Similarly to other conjugation systems described in this review, a negative feedback-loop exists that mediates shut-off of *tra* gene expression via the DNA binding protein TraY which has an activating role at low concentrations but can inhibit *tra* gene expression at higher concentrations. Other factors that contribute to the shut-off of *tra* gene transcription or modulate and fine tune this system are extracellular and cellular stress response elements, including the CpxAR two component system, proteases, and the chaperone protein GroEL (Zahrl et al., 2006, 2007; Lau-Wong et al., 2008).

## Population heterogeneity and conjugation in biofilms

From studies of many different conjugation systems it has become evident that, even under controlled laboratory conditions, the transition to transfer competence does not occur in all cells of a population (Figure 2B). One example in which this phenomenon has been illustrated nicely is the demonstration of discontinuous patches of gene transfer between donor and recipient cells at the edge of bacterial colonies on semi-solid agar surfaces (Reisner et al., 2012). Higher magnifications of these zones revealed that these patches correspond to infrequently occurring gene transfer events from some cells of the donor cell population to recipient cells but not from all. This phenomenon was observed in the case of a derivative of the naturally repressed F-like plasmid R1 with an intact FinOP repressor system (see above). These findings are consistent with the observation in liquid media where *tra* gene expression is low in the presence of plasmid R1 compared to a de-repressed mutant. Furthermore, gene transfer in liquid media occurs at a low frequency indicating that only about 1% of donor cells have activated their *tra* genes and transformed into transfer competent cells (Wagner et al., 2013).

Since the first observation published by Ghigo (2001) that the presence of CPs in bacterial populations induces the formation of biofilms it has become increasingly evident that these microbial communities are hot-spots for social interactions and horizontal gene transfer (HGT). In short, CPs promote biofilm formation and, vice versa, biofilms promote conjugation (Molin and Tolker-Nielsen, 2003; Madsen et al., 2012). The underlying gene regulatory mechanisms, however, are largely unknown because *tra* gene expression studies in biofilms are difficult to perform due to the dynamic nature of biofilms and the associated inherent heterogeneity of cells. Which donor cells in a biofilm community actually progress (via activation of *tra* gene expression) to transfer competent cells is unknown. What can be observed at the single cell level by sophisticated genetic constructs and fluorescence microscopy, however, is the transfer of plasmid DNA into recipient cells and the spread of the CP in the recipient population. In one case, the transfer of the pWW0 TOL plasmid from *Pseudomonas putida* donor to recipient microcolonies on semi-solid agar surfaces was investigated. Intriguingly, time-lapse microscopic images revealed that spreading of the CP in the recipient originated from one transfer event between cells contacting each other at the edges of donor and recipient microcolonies. This single transfer event was followed by limited, cell division dependent, spreading of the CP in the recipient colony. Again, similarly to plasmid R1 (see above), not all donor cells that were in contact with recipient cells initially transferred the CP, indicating regulatory mechanisms that maintain the OFF state in most of the cells of the donor microcolony (Seoane et al., 2011). Regulatory mechanisms including negative autoregulation by a transcriptional repressor of *tra* genes that could account for a shut-off after an initial burst have indeed been demonstrated for the pWW0 plasmid (Lambertsen et al., 2004). In analogy to the microcolony situation, limited invasion of recipient cells in *E. coli* biofilms has been demonstrated by a different method for a variety of conjugative antibiotic resistance plasmids (Król et al., 2013). Interestingly, in case of ICE*bs1*, rapid conjugative spreading of the ICE in recipient *B. subtilis* cell chains was observe and it has been suggested that such a mechanism can accelerate the spread of conjugative elements in microbial communities (Babic et al., 2011).

## Fitness cost of CPs and coevolution of CPs and host cells

In theory, CPs should, once established in a bacterial host, represent a burden and generate a fitness disadvantage, resulting eventually in the elimination of the plasmid from a population. This, however, as evidenced by the persistence of these elements, seems not to be the case. So how is the cost of DNA replication and CP gene expression kept low? Are there advantages conferred to the host by the CP in the absence of selection for genes that are carried by the CP? There are several studies in which the apparent paradox of the persistence of CPs in the bacterial world has been investigated (Modi and Adams, 1991; Dahlberg and Chao, 2003; Dionisio et al., 2005; Harrison and Brockhurst, 2012). One interesting result of such studies was that, CPs such as R1 and RP4 were not lost from bacterial populations even after more than 1000 generations of growth without selective pressure. This was attributed to the fact that such plasmids have a controlled replication system and low copy numbers as well as active partitioning and plasmid stability systems that prevent plasmid loss. An initial minimal fitness cost that was imposed on the *E. coli* host by R1 and RP4 (in comparison to plasmid free cells) was reduced or completely absent after 1100 generations. Coevolution induced changes were observed in both the evolved host cells and plasmids. Interestingly, evolved plasmid R1 had slightly lower transfer rates in the evolved host than in the ancestral host (Dahlberg and Chao, 2003). These findings were corroborated by a similar study where it was found that an evolved plasmid R1 even conferred a relative fitness advantage to the original *E. coli* host and a novel *Salmonella enterica* host. In addition, the original R1 plasmid had no fitness cost in the evolved *E. coli* strain suggesting coevolution of both the host cell and the CP (Dionisio et al., 2005). These results and other studies have led to the proposal that CP mediated bacterial conjugation is a coevolutionary process (Harrison and Brockhurst, 2012). Although not measured directly, the data of Dahlberg and Chao (2003) suggest that a major cost of CP carriage is the expression of *tra* genes. In line with this proposition is the fact that the activation of *tra* genes in case of plasmid R1 causes the up-regulation of extracytoplasmic and cytoplasmic stress regulons (Zahrl et al., 2006). In addition, F plasmid *tra* gene expression and T4S system assembly causes increased sensitivity to bile salts (Bidlack and Silverman, 2004). In any case, due to the regulatory regime that keeps *tra* genes OFF in the majority of donors, metabolic burden and exposure to pilus specific bacteriophages is not evenly distributed within a population but instead restricted to a small fraction of the population. In this way, possible detrimental and cell threatening effects associated with *tra* gene expression are limited to a few cells within a population whereas all cells retain beneficial genes and the potential for HGT. An intrinsically beneficial feature contributing to the persistence of CPs within bacterial populations may be their well documented ability to promote formation of biofilms (see above).

Interestingly, besides the specific regulatory mechanisms discussed in this review that operate to control *tra* gene expression, there is a general silencing mechanism in enterobacteria that mediates silencing of laterally acquired genes by H-NS and related proteins (Navarre et al., 2007). This repression of foreign genes termed “xenogeneic silencing” was also found in plasmid F (Will and Frost, 2006) and the naturally repressed plasmid R1 where H-NS contributes to the low frequency of transfer competent cells in a population of cells carrying this CP (Wagner et al., 2013).

## Conclusion and outlook

Although the molecular details of how regulatory networks control *tra* gene expression are different in the conjugation systems presented in this review, there is a common theme: As a default, *tra* genes are OFF and whenever positive stimuli are present, not the whole population transits to the ON stage but only a fraction of the cells carrying a conjugative element. In this way the metabolic burden (fitness cost) imposed by expression of *tra* genes and assembly of a cell envelope localized DNA secretion machine (a T4SS) is carried not by the whole population but distributed to only a few cells within a population. Further studies at the single cell level are needed to reveal whether the transformation of only a fraction of a donor cell population into transfer competent cells is due to a stochastic process or depends on different physiological states such as metabolic conditions, cellular fitness and cell age. Moreover, positioning of individual cells in structured communities (microcolonies or biofilms) may influence transition to transfer competence.

Undoubtedly intelligent strategies exist to minimize or even eliminate fitness costs associated with the carriage of conjugative elements. Populations harboring CPs (and presumably ICEs) can grow and divide largely unaffected by the presence of these elements. At the same time, some cells within a population do become transfer competent and thereby secure the spread and persistence of conjugation modules in many different bacterial species, among them pathogens causing disease in humans, animals, and plants. Thus, genes carried on the conjugative element, which are beneficial for the host cell in particular habitats (e.g., antibiotic resistance genes), are likely to persist in bacterial populations even without continuous selective pressure.

### Conflict of interest statement

The authors declare that the research was conducted in the absence of any commercial or financial relationships that could be construed as a potential conflict of interest.
